# Serum lipoprotein levels in takotsubo cardiomyopathy vs. myocardial infarction

**DOI:** 10.1186/1755-7682-4-14

**Published:** 2011-04-28

**Authors:** Sainath Gaddam, Krishna C Nimmagadda, Tarun Nagrani, Muniba Naqi, Robert V Wetz, Kera F Weiserbs, Donald McCord, Foad Ghavami, Bhavesh Gala, James C Lafferty

**Affiliations:** 1 Department of Internal Medicine, Staten Island University Hospital, New York, USA; 2 Division of Cardiology, Massachusetts General Hospital, Massachusetts, USA; 3 Department of Academic Affairs, Staten Island University Hospital, New York, USA; 4 Division of Cardiology, Staten Island University Hospital, New York, USA

## Abstract

**Background:**

In the setting of myocardial infarction (MI) or acute coronary syndrome (ACS), current guidelines recommend early and aggressive lipid lowering therapy with statins, irrespective of the baseline lipoprotein levels. Takotsubo cardiomyopathy (TCM) patients have a clinical presentation similar to myocardial infarction and thus receive early and aggressive statin therapy during their initial hospitalization. However, the pathology of TCM is not atherosclerotic coronary artery disease and hence we assumed the lipid profiles in TCM would be healthier than coronary artery disease patients.

**Methods:**

In this retrospective study, we assessed fasting serum lipoprotein levels of ten TCM patients and compared them with forty, age and sex-matched myocardial infarction (MI) patients.

**Results:**

Comparing serum lipoprotein levels of TCM with MI group, there was no significant difference in mean total cholesterol between the two groups (174.5 mg/dL vs. 197.6 mg/dL, p = 0.12). However, in the TCM group, mean HDL-C was significantly higher (66.87 mg/dL vs. 36.5 mg/dL, p = 0.008), the mean LDL-C was significantly lower (89.7 mg/dL vs. 128.9 mg/dL, p = 0.0002), and mean triglycerides was also significantly lower (65.2 mg/dL vs. 166.8 mg/dL, p < 0.0001).

**Conclusions:**

In this study, TCM patients in comparison to MI patients had significantly higher levels of HDL-C, lower levels of LDL-C levels and triglycerides. The lipid profiles in TCM were consistent with the underlying pathology of non-atherosclerotic, non-obstructive coronary artery disease. As lipoproteins in most TCM patients were within the optimal range, we recommend an individual assessment of lipid profiles along with their coronary heart disease risk factors for considering long term lipid-lowering therapy. A finding of hyperalphalipoproteinemia or hypotriglyceridemia in 40% of TCM patients is novel but this association needs to be confirmed in future studies with larger sample sizes. These findings may provide clues in understanding the pathogenesis of takotsubo cardiomyopathy.

## Background

Takotsubo cardiomyopathy (TCM) is gaining increasing popularity due to its heightened awareness among western nations [1]. Emotional or physical stress, leading to catecholamine surge causing myocardial stunning is the most widely accepted mechanism [2]. Presenting symptoms of TCM are similar to acute coronary syndrome (ACS) with the vast majority complaining of chest pain, associated with electrocardiographic changes, mild elevation of cardiac biomarkers, "octopus pot/apical ballooning" appearance on ventriculogram, and non-obstructive coronary arteries. The reversal of cardiomyopathy in a few days to a few weeks is a characteristic feature [3].

Most patients with TCM have a favorable prognosis and the treatment is geared towards managing systolic heart failure by using aspirin, diuretics, beta-blockers and ACE inhibitors. Our electronic search in the MEDLINE database (accessed via PUBMED) using a detailed search strategy found only one retrospective study comparing medical therapy and outcomes in TCM patients [4]. This study by Fazio et al in 2007 suggested there was no benefit of long term therapy in TCM patients by administering either aspirin, diuretics, beta-blockers, ACE inhibitors or calcium channel blockers [4]. However, this study did not analyze the role of lipid-lowering therapy in TCM patients.

In the setting of ACS, early and aggressive statin therapy is beneficial [5]. Since TCM patients have a presentation similar to ACS, they are started on aggressive statin therapy upon hospitalization. We presumed, since the underlying pathology in TCM is not atherosclerotic in nature, the lipid panels in TCM patients would be in the optimal range and would differ from those with atherosclerotic coronary heart disease.

## Methods

We retrospectively evaluated patients diagnosed in our hospital with TCM and myocardial infarction (MI) between January 2005 and April 2010. In this five year period, a total of fourteen patients were diagnosed with TCM. Out of these fourteen patients, four were excluded from the study because two patients were diagnosed based on echocardiographic findings without a diagnostic coronary angiogram and two others did not have an in-hospital lipoprotein level measurement. We compared lipoprotein levels of the remaining ten TCM patients with forty randomly selected MI patients (both ST and non-ST elevation MI), who were matched by age and gender.

Lipoprotein levels were retrospectively retrieved from patient records. The coronary angiograms of all selected subjects were reviewed independently by two cardiologists. TCM patients were confirmed to have non-obstructive coronary arteries (< 50% diameter stenosis) with typical apical and/or mid-ventricular wall motion abnormalities. All patients in the TCM group had complete recovery of cardiomyopathy, evidenced by clinical improvement and assessment of left ventricular function using transthoracic echocardiogram. The patients in the MI group had angiographic evidence of severe obstructive coronary artery disease (> 50% diameter stenosis) and underwent revascularization procedures.

Hypertension, diabetes, peripheral arterial disease, and abdominal aortic aneurysm were defined by a history or treatment of these conditions. Dyslipidemia was defined by a history of abnormal cholesterol (total cholesterol > 200 mg/dL, HDL < 40 mg/dL, LDL > 130 mg/dL, or triglycerides > 200 mg/dL) or currently taking any lipid-lowering medications. Smoking history was considered positive if they had ever smoked in their lives. Family history of premature coronary artery disease (first-degree relative < 55 years in men, < 65 years in women) was obtained. Body mass index (BMI) was calculated based on height and weight. The institutional review board (IRB) of Staten Island University Hospital approved the protocol.

## Results

### Clinical characteristics of TCM patients

In the TCM group, eighty percent of the population were women and were all postmenopausal. The mean age of patients was 62 years (range, 54 to 68 years) and mean BMI was 23.9 kg/m2 (range, 19.1 kg/m2 to 31.1 kg/m2). The most common presenting complaint to the hospital was chest pain (90%) and in a few patients was associated with shortness of breath (20%), syncopal episode (10%) or palpitations (10%). Most patients had either two or more coronary risk factors, but none had a prior history of coronary artery disease or coronary heart disease equivalents. All patients in the TCM group were non-diabetics and their mean blood glucose level at the time of admission (non-fasting) was 121.4 mg/dL. The most common electrocardiographic findings at presentation were prolonged corrected QT intervals (60%) and ST elevations (50%). The average peak troponin I elevation was 3.2 ng per milliliter (normal value < 0.03 ng per milliliter) and the average peak creatine kinase MB level was 21.09 ng per milliliter (normal value < 5.0 ng per milliliter). Severe left ventricular dysfunction was present in all TCM patients with the mean ejection fraction at presentation being 30%.

Clinical characteristics have been described in Table 1.

**Table 1:** Clinical characteristics of Takotsubo cardiomyopathy patients

**Patient No**.	AGE	SEX	RACE	TC	LDL	HDL	TRIG	VLDL	LVEF%
1	65	F	C	221	76	135	50	9	30
2	65	F	C	235	116	93	113	22	25
3	64	F	C	170	73	66	36	6	35
4	61	F	C	177	96	64.5	82	14	25
5	65	F	AA	162	90	65	34	6	35
6	54	F	C	156	73	61	71	12	20
7	60	M	C	145	76	56	63	11	25
8	60	M	C	190	121	53	81	14	30
9	68	F	C	174	107	46	64	12	45
10	61	F	C	115	69	30	58	11	30

C - CAUCASIAN, AA - AFRICAN AMERICAN, TC - TOTAL CHOLESTEROL, TRIG - TRIGLYCERIDES, LVEF%- LEFT VENTRICULAR EJECTION FRACTION AT PRESENTATION

### Lipoprotein Levels in TCM vs. MI patients

In the TCM group, the mean total cholesterol was 174.5 mg/dL (range 115-235 mg/dL), the mean HDL-C level was 66.87 mg/dL (range 30-135 mg/dL), the mean LDL-C level was 89.7 mg/dL (range 69-121 mg/dL), and mean triglyceride level was 65.2 mg/dL (range 34-113 mg/dL). All TCM patients had an LDL-C < 130 mg/dL and only one patient had an HDL-C < 40 mg/dL. Two other patients had unusually high levels of HDL-C of 93 and 135 mg/dL, which are in the range of hyperalphalipoproteinemia. Two other patients had mild hypotriglyceridemia with triglyceride levels of 34 and 36 mg/dL.

In the MI group, the mean total cholesterol was 197.6 mg/dL (range 93-363 mg/dL), mean HDL-C was 36.5 mg/dL (range 18-61 mg/dL), mean LDL-C was 128.9 mg/dL (range 41-232 mg/dL) and mean triglyceride level was 166.9 mg/dL (range 46-388 mg/dL).

Comparing serum lipoprotein levels of the TCM group with the MI group, there was no significant difference in mean total cholesterol between the two groups (174.5 mg/dL vs. 197.6 mg/dL, p = 0.12). However, in the TCM group, mean the HDL-C was significantly higher (66.87 mg/dL vs. 36.5 mg/dL, p = 0.008), the mean LDL-C was significantly lower (89.7 mg/dL vs. 128.9 mg/dL, p = 0.0002), and the mean triglyceride level was significantly lower (65.2 mg/dL vs. 166.8 mg/dL, p < 0.0001).

## Discussion

The main results of our study are: (a) The TCM group had significantly higher HDL-C (p = 0.008), lower LDL-C (p = 0.0002) and lower triglycerides (p < 0.0001) compared to age and sex-matched MI patients; and (b) Hyperalphalipoproteinemia or mild hypotriglyceridemia was noted in 40% of TCM patients.

The American Heart Association (AHA) and Adult Treatment Panel (ATP Ш) guidelines recommend a target LDL-C of less than 130 mg/dL in patients with two or more coronary risk factors and have no evidence of coronary heart disease or coronary heart disease equivalents [6]. In our study, most patients with TCM had two or more coronary heart disease risk factors, but their mean LDL-C was 89.7 mg/dL (range 69-121 mg/dL), mean HDL-C was 66.87 mg/dL and mean triglycerides was 65.2 mg/dL, which is considered optimal by AHA/ATP Ш guidelines. In the MI group, the mean LDL-C was 128.9 mg/dL and mean HDL-C was 36.5 mg/dL, these findings of elevated of LDL-C and low HDL-C are consistent with prior studies on myocardial infarction and support the need for early and aggressive lipid-lowering therapy [7]. Multiple studies on TCM have established a non-atherosclerotic, non-obstructive coronary artery disease pattern and our study supports these findings by demonstrating the existence of a healthy lipid panel in this population.

Hypotriglyceridemia is a rare entity that can occur in severe malnutrition and some autoimmune diseases. In one research study, hypotriglyceridemia was shown to be a precocious marker for autoimmunity [8]. Though TCM-like autoimmune diseases is predominant in female population, currently there is no evidence to support an autoimmune component in TCM pathogenesis. None of our patients had a history or symptoms suggestive of autoimmune diseases. Hyperalphalipoproteinemia was noted in two other TCM patients, a term used when HDL-C levels are more than 90 mg/dL. The major function of HDL-C is reverse cholesterol transport, during which, peripherally deposited cholesterol is transferred to the liver. High HDL-C is protective [9] and is associated with low VLDL and low triglycerides, as observed in our patients in TCM group. Metabolic abnormality has been suggested to play a role in TCM pathogenesis, whether these findings play a role in the inverse metabolic-perfusion mismatch observed in nuclear imaging studies needs further investigation [10-12].

Our observational study, like most studies on TCM, has a major limitation of small sample size due to the rarity of the disease. Also, there is a controversy on assessing serum lipid levels during hospitalization, due to the wide range of variability noted with the in-hospital and 1-2 months post-discharge lipoprotein measurement [13-15]. It is postulated that in acute coronary syndrome, serum triglycerides act as acute phase reactants and its variability with fasting states alters the assessment of serum lipoprotein measurements [16]. However, recent trials with large sample sizes concluded that mean serum LDL-C and other lipoproteins vary minimally in the early days of hospitalization and are reliable enough to start lipid-lowering therapy [17].

## Conclusion

In conclusion, the lipid panels in TCM were significantly healthier and different from the MI group and add to the evidence that the pathogenesis of TCM is not due to atherosclerotic blockage of epicardial coronary arteries. Long term lipid lowering therapy in TCM should be individualized based on the coronary risk factors and their serum lipoprotein levels. In this single center experience, TCM patients had significantly higher HDL-C levels, and lower LDL-C levels and triglyceride levels in comparison to the MI group.

## List of abbreviations

TCM: takotsubo cardiomyopathy; MI: myocardial infarction; ACS: acute coronary syndrome; LDL-C: low density cholesterol; HDL-C: high density cholesterol; VLDL: very low density cholesterol; TRIG: triglycerides

## Conflict of Interests

The authors declare that they have no competing interests.

## Authors' contributions

SG designed the study, collected data, wrote the manuscript and was responsible for the intellectual input. KCN, TN, MN helped with the literature search. KW was involved in the statistical analysis. RVW, FG, DM, BG, JCL were involved in reviewing the manuscript. All authors read and approved the final manuscript.

## Consent

Written informed consent was obtained from the patient for publication of this case report and accompanying images. A copy of the written consent is available for review by the Editor-in-Chief of this journal.
